# Recent Advances of Bimetallic Sulfide Anodes for Sodium Ion Batteries

**DOI:** 10.3389/fchem.2020.00353

**Published:** 2020-05-06

**Authors:** Yu Huang, Dongbin Xiong, Xifei Li, Hirbod Maleki Kheimeh Sari, Jianhong Peng, Yingying Li, Yunyan Li, Dejun Li, Qian Sun, Xueliang Sun

**Affiliations:** ^1^Tianjin International Joint Research Center of Surface Technology for Energy Storage Materials, College of Physics and Materials Science, Tianjin Normal University, Tianjin, China; ^2^Xi'an Key Laboratory of New Energy Materials and Devices, Institute of Advanced Electrochemical Energy & School of Materials Science and Engineering, Xi'an University of Technology, Xi'an, China; ^3^State Center for International Cooperation on Designer Low-carbon & Environmental Materials (CDLCEM), Zhengzhou University, Zhengzhou, China; ^4^School of Physical and Electronic Information Engineering, Qinghai Nationalities University, Xining, China; ^5^Department of Mechanical and Materials Engineering, University of Western Ontario, London, ON, Canada

**Keywords:** energy storage systems, lithium ion batteries, sodium ion batteries, bimetallic sulfides, anode materials

## Abstract

The high usage for new energy has been promoting the next-generation energy storage systems (ESS). As promising alternatives to lithium ion batteries (LIBs), sodium ion batteries (SIBs) have caused extensive research interest owing to the high natural Na abundance of 2.4 wt.% (vs. 0.0017 wt.% for Li) in the earth's crust and the low cost of it. The development of high-performance electrode materials has been challenging due to the increase in the feasibility of SIBs technology. In the past years, bimetallic sulfides (BMSs) with high theoretical capacity and outstanding redox reversibility have shown great promise as high performance anode materials for SIBs. Herein, the recent advancements of BMSs as anode for SIBs are reported, and the electrochemical mechanism of these electrodes are systematically investigated. In addition, the current issues, challenges, and perspectives are highlighted to address the extensive understanding of the associated electrochemical process, aiming to provide an insightful outlook for possible directions of anode materials for SIBs.

## Introduction

In the past years, fossil fuels have been overexploited as the primary source of energy for industries and people's daily lives around the world. Simultaneously, risks of the resource shortage and the pollution of the environment due to the burning of fossil fuels have caused a development in the research and application of renewable energy sources. In the early 1990s, LIBs became an essential power supply in a variety of electronic devices since their first commercialization by Sony. With the increasing demand of clean energy, LIB has been one of the most indispensable energy storage technologies (Maleki Kheimeh Sari and Li, 2019; Su et al., 2020). However, the limited lithium resources and the high cost of Li have hampered the large-scale applications of LIBs. Therefore, it is of great importance to explore a new and novel candidate as an alternative for this type of batteries (Che et al., 2017; Hwang et al., 2017; Kang et al., 2017; Ortiz-Vitoriano et al., 2017; Xiao et al., 2017; Fan and Li, 2018).

In recent years, SIBs have attracted much attention due to the similarities between Na and Li in terms of the chemical/electrochemical properties. Besides, sodium is the fourth most abundant metal element after aluminum, iron and calcium, which is evenly distributed in the earth's crust (Yu and Chen, 2020). Additionally, due to the abundant and cheap resources of Na, SIBs were considered as one of the most promising candidates for large-scale renewable energy storage systems to store electricity from solar, wind and waves (Palomares et al., 2012; Kim et al., 2015; Kundu et al., 2015; Fan et al., 2016). However, there are still many differences between these two elements. As shown in Table 1, sodium has a larger ionic radius (1.02 Å) than that of Li (0.76 Å) which is heavier than an atom, and also a higher standard electrode potential (Slater et al., 2013; Chen J. et al., 2017; Meng, 2017; Xiao et al., 2017; Fang Y. et al., 2018; Wang et al., 2018). Although SIBs are inferior to LIBs in terms of energy density and charge-discharge rate, Li and Na are just a fraction of the whole electrode, and the capacity is largely depended on the characteristics of the active materials. Therefore, exploring exceptional properties anodes for advanced SIBs is the key point in developing this technology, which is indeed accompanied with many challenges (Li and Wang, 2012; Cao et al., 2017; Lin et al., 2018; Xiong et al., 2018). In general, a well-designed nanostructure materials can shorten ion diffusion paths and electron, routes, and mitigate the mechanical stress caused by large volume expansion. Additionally, comparing to the carbon-based anode materials (e.g., porous carbon, nitrogen-doped carbon nanofibers) (Lai et al., 2012; Kong et al., 2014; Xiao et al., 2014, 2017), metallic compound materials possess a higher theoretical specific capacity due to their excellent electrochemical conversion mechanism (Yang et al., 2015; Yu et al., 2015; Chen Y. et al., 2016; Wu et al., 2016; Yu X-Y. et al., 2016; Wen et al., 2017). For example, many mono-layered transition metal oxides (MOs-NiO_2_, FeO_2_, TiO_2_, MnO_2_, and etc Xia et al., 2014; Yu D. J. et al., 2016) have been extensively studied as Na storage materials. NiO_2_ exhibited a reversible capacity of about 123 mAh g^−1^ with a small polarization. Mono-layered FeO_2_ showed the largest reversible capacity (up to 80 mAh g^−1^) at a high cut-off voltage of 3.5 V. When used as electrode material in SIBs, TiO_2_ also showed an excellent capacity retention (25% capacity fading over 1,200 cycles). Indeed, MnO_2_ was synthesized by a simple redox reaction and hydrothermal treatment method, and a large discharge capacity of 219 mAh g^−1^ was delivered. Jiang et al. developed a Fe_2_O_3_ thin film as an anode for SIBs with a steady capacity of 380 mAh g^−1^ after 200 cycles. However, metallic oxides (MOs) have several disadvantages derived from their low electroconductibility and electrochemical activity (Du et al., 2015; Zhu et al., 2015; Yu and David Lou, 2018).

**Table 1:** Comparison of Li and Na.

	**Li**	**Na**
Ionic radius	0.76 Å	1.02 Å
Content in the earth's crust/Reserve	0.0065%/1350W t	2.4%/500,000 W t
Cost of trona (per ton)	$5,000	$135–165
Weight	7 g mol^−1^	23 g mol^−1^
Standard electrode potential (vs. SHE)	−3.02 V	−2.71 V

Among various anode materials reported for SIBs, metallic sulfides (MSs) have drawn much attention due to their reversibility of redox reactions, excellent capacity and faster conductivity compared to MOs. M-S bond in MSs is weaker than homologous M-O bond in MOs due to the different electronegativity of S and O, facilitating chemical reactions during the charge-discharge (Li et al., 2015; Yu X-Y. et al., 2016; Zheng et al., 2017). For example, MoS_2_ nanosheets as anode material in SIBs showed a good charge-discharge capacity of 386 mAh g^−1^. However, MSs suffer from severe problems such as volume expansion during the Na^+^ insertion/extraction process, sluggish Na^+^ diffusion kinetics, and poor electrical conductivity, which may result in some defects accompanied by capacity fading, poor cycle life, and unacceptable rate performance. Many researches have been known to enhance the electrochemical performance of these anode materials through reasonable structural design (Zhou Q. et al., 2016; Hwang et al., 2017).

Along with MSs, BMSs have also become a hot topic as SIBs anode materials regarding their high electronic conductivity, good electrochemical activity, and strong electrochemical controllability (Li et al., 2013; Youn et al., 2016; Li Y. et al., 2017; Tang et al., 2017). Thus far, BMSs with different morphologies and structures, (e.g., nanosheets, nanoplates, nanotubes, ball-in-ball hollow spheres, nanopetals, and urchin-like structures) have been reported as high performance anodes in LIBs (Chen T. et al., 2016; Li et al., 2016; Ma et al., 2016). Up to now, there are quite a number of remarkable works in regards to the application of BMSs as anode materials in LIBs. The synergistic effect between BMSs with higher theoretical capacity and optimized nanostructure can more effectively maintain the mechanical stability compared with MOs and MSs (Lai et al., 2012; Kong et al., 2014; Chen Y. et al., 2016; Wu et al., 2016). One example is 0D/1D C@FeCo-S NDS/CNR composite prepared through hydrothermal method (Gao et al., 2017), or yolk–shell-structured Fe–Ni–S powders and (Ni_0.3_Co_0.7_)_9_S_8_/N-CNTs/rGO composite with ultrahigh long-life cycling stability and outstanding rate property as an anode for SIBs. The reason may be owing to their smaller volume change and higher initial coulombic efficiency (ICE), yielding a low irreversible capacity (Kim and Kang, 2017). Li and his co-workers prepared NiCo_2_S_4_ with N-doped carbon served as an anode material for SIBs through a bottom-up strategy, and by adjusting optimal voltage region an outstanding capacity of 570 mAh g^−1^ over 200 cycles at 0.2 A g^−1^ was obtained (Li S. et al., 2019).

Moreover, BMSs possess higher electronic conductivity and more abundant redox reactions than single MSs, which can dramatically strengthen the electrochemical performances. However, there are only a few reviews concentrated on BMS-based anodes for SIBs (Yan et al., 2014; Fan et al., 2016; Chang et al., 2017). The recent progresses of BMS anode in SIBs, the various synthesis strategies, and their sodium storage mechanisms along with their limitations are systematically discussed in this review. In the end, the existing challenges and opportunities for designing high-performance BMS anodes for SIBs are introduced.

## Sodium Storage Mechanism

Owing to the high theoretical specific capacity and low cost, BMSs have been a propitious class of anode materials for both LIBs and SIBs (Duan et al., 2019). When used in SIBs, BMSs can reserve Na^+^ via special mechanism. In some cases, an intercalation/de-intercalation process or an alloy-dealloying reaction happens in the process of charge-discharge, which is depended on BMSs (Li Z. et al., 2017; Yan et al., 2017).

Generally, in the first discharge process of BMSs (e.g., NiCo_2_S_4_ (Zhang et al., 2018), CuCo_2_S_4_ (Gong et al., 2018; Li Q. et al., 2019), Ti_0.25_Sn_0.75_S_2_ (Huang et al., 2018), and ZnSnS_3_ Jia et al., 2018; Liu et al., 2019), Na^+^ intercalates into BMSs and then a reversible conversion reaction occurs (Li S. et al., 2019). The principle of correlation reaction is similar to that of LIBs. Nonetheless there are some distinctions in the reaction process between SIBs and LIBs (Stephenson et al., 2014; Zhang et al., 2014). The first reduction process is attributed to Na^+^ intercalation in BMSs without any phase transformation, Equation (1). In the same cycle the conversion reactions occur, as summarized in Equations (2) and (3), which provide an impressive capacity cause structural instability (Jin et al., 2015; Song et al., 2017; Li S. et al., 2019).

(1) $$\begin{array}{l} {\text{MS}}_{\text{x}}+\text{xN}{\text{a}}^{+}+\text{x}{\text{e}}^{-}\to {\text{Na}}_{\text{x}}{\text{MS}}_{\text{x}} \end{array}$$

(2) $$\begin{array}{l} \text{N}{\text{a}}_{\text{x}}\text{M}{\text{S}}_{\text{x}}+\left(2-\text{x}\right)\text{N}{\text{a}}^{+}+\left(2-\text{x}\right){\text{e}}^{-}\to \text{MS}+\text{N}{\text{a}}_{2}\text{S} \end{array}$$

(3) $$\begin{array}{l} \text{MS}+2\text{N}{\text{a}}^{+}+2{\text{e}}^{-}\to \text{M}+\text{N}{\text{a}}_{2}\text{S} \end{array}$$

As another kind of Na storage mechanism, ZnSnS_3_ is used as an anode for SIBs, Na^+^ intercalates into the layered structure in the initial sodiation process. During the whole electrochemical process, a combined conversion mechanism and alloy-dealloying mechanism occurs. The corresponding reaction can be depicted as follows (e.g., ZnSnS_3_): (Fu et al., 2015; Qin et al., 2016b; Dong et al., 2017; Deng et al., 2018; Zhang Y. et al., 2019).

(4) $$\begin{array}{r} \text{Conversion reaction}:\text{ZnSn}{\text{S}}_{3}+6\text{N}{\text{a}}^{+}+6{\text{e}}^{-}\\ \to \text{Sn}+\text{Zn}+3\text{N}{\text{a}}_{2}\text{S} \end{array}$$

(5) $$\begin{array}{l} \text{Alloying reaction}:4\text{Sn}+13\text{Zn}+16\text{N}{\text{a}}^{+}+16{\text{e}}^{-}\\ \to \text{N}{\text{a}}_{15}\text{S}{\text{n}}_{4}+\text{NaZ}{\text{n}}_{13} \end{array}$$

It is important noting that during the electrochemical process of BMS electrodes (M = Zn, Co) conversion reactions are bound to happen and the following reaction equations can be speculated, NiCo_2_S_4_ can be used as an example, while Na_x_MS_y_ is the intermediate product of the intercalation reaction:

(6) $$\begin{array}{l} \text{Discharge}:\text{M}{\text{S}}_{\text{x}}+\text{xN}{\text{a}}^{+}+\text{x}{\text{e}}^{-}\to \text{N}{\text{a}}_{\text{x}}\text{M}{\text{S}}_{\text{x}}\left(\text{M}=\text{Ni}/\text{Co}\right)\\ 3.0-1.3\text{V} \end{array}$$

(7) $$\begin{array}{l} \text{N}{\text{a}}_{\text{x}}\text{M}{\text{S}}_{\text{x}}+\left(2-\text{x}\right)\text{N}{\text{a}}^{+}+\left(2-\text{x}\right){\text{e}}^{-}\to \text{MS}+\text{N}{\text{a}}_{2}\text{S}\\ 1.3-0.6\text{V} \end{array}$$

(8) $$\begin{array}{l} \text{MS}+2\text{N}{\text{a}}^{+}+2{\text{e}}^{-}\to \text{M}+\text{N}{\text{a}}_{2}\text{S}\\ 0.6-0.1\text{V} \end{array}$$

(9) $$\begin{array}{l} \text{NiC}{\text{o}}_{2}{\text{S}}_{4}+8\text{N}{\text{a}}^{+}+8{\text{e}}^{-}\to 4\text{N}{\text{a}}_{2}\text{S}+\text{Ni}+2\text{Co}\\ 3.0-0.1\text{V} \end{array}$$

(10) $$\begin{array}{l} \text{Charge}:\text{Ni}+\text{N}{\text{a}}_{2}\text{S}\to \text{Ni}{\text{S}}_{\text{x}}+2\text{Na}\\ 0.1-0.7\text{V} \end{array}$$

(11) $$\begin{array}{l} \text{Co}+\text{N}{\text{a}}_{2}\text{S}\to \text{Co}{\text{S}}_{\text{x}}+2\text{Na}\\ 1.7-3.0\text{V} \end{array}$$

(12) $$\begin{array}{l} 2\text{N}{\text{a}}_{2}\text{S}+\text{Ni}+\text{Co}\to \text{Ni}{\text{S}}_{\text{x}}+\text{Co}{\text{S}}_{\text{x}}+4\text{Na}\\ 0.1-3.0\text{V} \end{array}$$

## Synthesis of BMS With Nanostructures

### Solvothermal Methods

As a low-cost and environmentally friendly synthetic method, solvothermal reaction is effective to synthesize various nanomaterials with disparate morphologies, complete crystal particles, small particle sizes, uniform distribution, controllable stoichiometry, and high crystallinity. Due to the above merits, the solvothermal method has been widely employed in synthesizing new structures and materials. In the past decades, this method has been frequently used in preparing oxide-based and S-based materials with ideal structure and controllable size for SIBs. Over recent years, BMSs with various morphologies have been successfully synthesized via solvothermal method. For example, NiCo_2_S_4_ nanodots with N-doped carbon (NiCo_2_S_4_@NC) (Li S. et al., 2019), NiCo_2_S_4_ hollow prism wrapped in reduced graphene oxide (rGO) (Zhang et al., 2018), N/S-rGO@ZnSnS_3_ amorphous ZnSnS_3_@rGO (Liu et al., 2019), ((Ni_0.3_Co_0.7_)_9_S_8_/N-CNTs/rGO) (Lv et al., 2018), (Co_0.5_Ni_0.5_)_9_S_8_/N-C) nanoparticles (Cao et al., 2019), CuCo_2_S_4_/rGO nanoparticles (Li Q. et al., 2019), and so forth. These nanostructured materials synthesized through solvothermal method possess strong controllability, excellent electrochemical performance, fast ions, and electron transfer paths and outstanding rate capability (Zhao and Manthiram, 2015; Liu et al., 2017; Jia et al., 2018; Chen et al., 2019).

**Figure 1:**
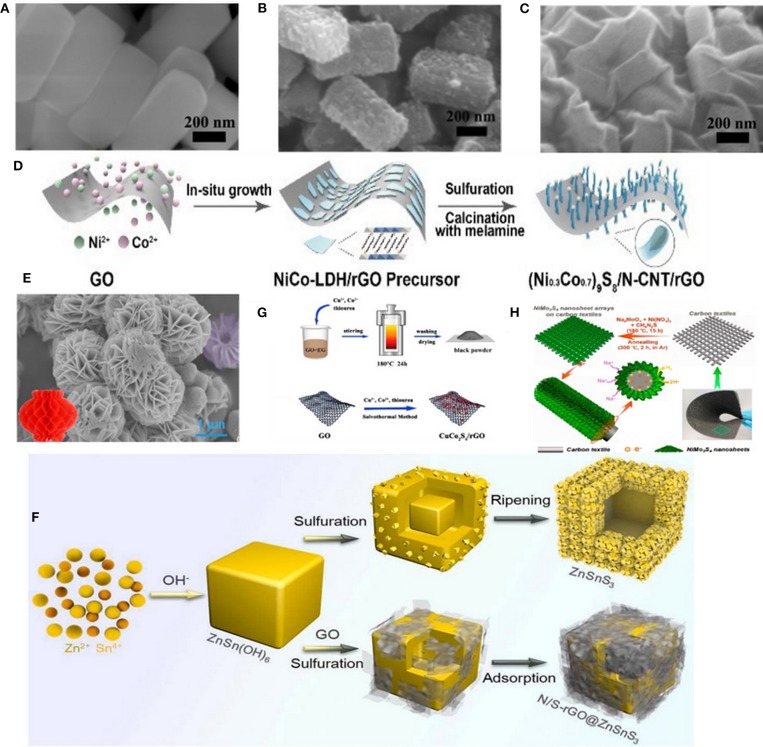
**(A–C)** SEM images of the NiCo precursor, NiCo_2_S_4_ and rGO-NiCo_2_S_4_, respectively. Reproduced with permission from Zhang et al. (2018) Copyright 2018, Royal Society of Chemistry. **(D)** Schematic illustration of preparing (Ni_0.3_Co_0.7_)_9_S_8_/N-CNTs/rGO. Reproduced with permission from Lv et al. (2018) Copyright 2018, Royal Society of Chemistry. **(E)** SEM images of the lantern-like Ti_0.25_Sn_0.75_S_2_ micro-particles. Reproduced with permission from Huang et al. (2018) Copyright 2018, Elsevier. **(F)** Schematic illustration for the preparation process of ZnSnS_3_ and N/S-rGO@ZnSnS_3_. Reproduced with permission from Liu et al. (2019) Copyright 2019, Elsevier. **(G)** Schematic illustration for the formation of CuCo_2_S_4_/rGO. Reproduced with permission from Gong et al. (2018) Copyright 2018, Elsevier. **(H)** Schematic illustration for the synthesis of 3D hierarchical NiMo_3_S_4_ nanosheet arrays on the flexible carbon textiles. Reproduced with permission from Kong et al. (2018) Copyright 2018, Elsevier.

A new type of hierarchical rGO wrapped NiCo_2_S_4_ composite was synthesized through refluxing and solvothermal reactions by Yin's group. As shown in Figures 1A–C, SEM images indicate that NiCo_2_S_4_ nanoprisms with a uniform size are tightly absorbed onto the negatively charged graphene oxide nanosheets because of the electrostatic interactions between them (Zhang et al., 2018). (Ni_0.3_Co_0.7_)_9_S_8_/N-CNTs/rGO nanoparticles were also obtained through a tightly *in-situ* growth onto rGO, as shown in Figure 1D (Lv et al., 2018). Chen et al. synthesized the lantern-like architecture Ti_0.25_Sn_0.75_S_2_ with MWCNTs composite through the hydrothermal method (Figure 1E) (Huang et al., 2018). Particularly, the unique architecture with abundant pores and large surface area can not only shorten the transmission path of Na^+^, but also reserve a large space for volume expansion. Liu's group, for the first time, designed hollow ZnSnS_3_ nano-microcubes with a N/S dual-doped rGO encapsulated (donated as N/S-rGO@ZnSnS_3_). During the preparation process, the precursor of ZnSn(OH)_6_ cubes have been successfully synthesized through an easy co-precipitation method. Afterwards, the precursor was mixed with Na_2_S, thiourea and GO dispersion, and finally the N/S-rGO@ZnSnS_3_ material was obtained through a typical hydrothermal reaction (Figure 1F) (Liu et al., 2019). The above-mentioned experiments were all following a two-step method. Nonetheless, recently, CuCo_2_S_4_/rGO nanocomposites was prepared through one-step solvothermal method by Zhao's group, as illustrated schematically in Figure 1G (Gong et al., 2018). Yang et al. also synthesized a bind-free SIBs anode material with hierarchical hybrid nanostructure which consisted of NiMo_3_S_4_ nanosheet arrays grown on flexible carbon textiles (denoted as NiMo_3_S_4_/CTs) through a one-step hydrothermal method and subsequent post-annealing process (Figure 1H) (Kong et al., 2018).

Moreover, VMo_2_S_4_-rGO nanosheets (Zhang K. et al., 2019), ZnSnS_3_@rGO nanoparticles (Jia et al., 2018), Cu_2_MoS_4_ nanoparticles (Chen et al., 2019), CuCo_2_S_4_ sub-microspheres (Li Q. et al., 2019), and CoSnS_x_@NC nanoboxes (Liu et al., 2017) have been successfully prepared using a similar approach.

### Spray Pyrolysis

Spray pyrolysis is a popular method for preparing BMSs with small particle size and good dispersion. Indeed, spray pyrolysis is a processing technique being considered in many researches to prepare thin and thick films, ceramic coatings, and powders. It offers an extremely easy approach for preparing samples of any composition. Compared with other deposition techniques, spray pyrolysis presents a very simple and relatively low-cost processing way.

**Figure 2:**
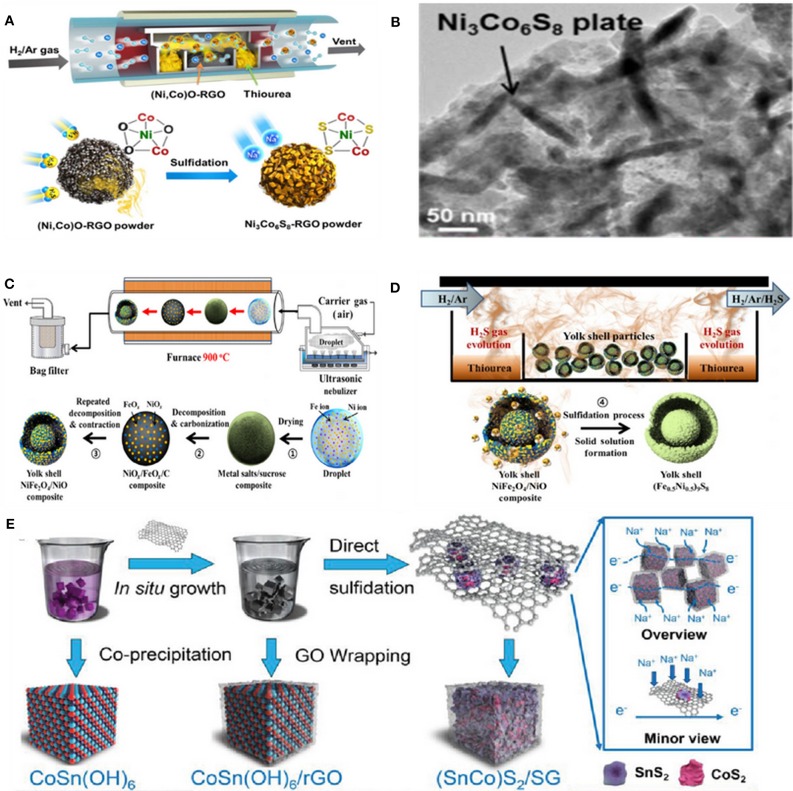
**(A)** Schematic illustration for the formation mechanism of the Ni_3_Co_6_S_8_-rGO powder. **(B)** TEM image of the Ni_3_Co_6_S_8_-rGO composite powder. Reproduced with permission from Choi and Kang (2015a) Copyright 2015. Royal Society of Chemistry. **(C)** Schematic diagrams for the preparation of the carbon-free Fe–Ni–O powders **(D)** the sulfidation process. Reproduced with permission (Kim and Kang, 2017) Copyright 2017, Tsinghua University Press. **(E)** Schematic illustration for the synthesis of (SnCo)S_2_/SG. Reproduced with permission from Yang et al. (2019) Copyright 2019. Wiley-VCH.

For instance, a hollow Ni_3_Co_6_S_8_-rGO sphere with plate-shape nanocrystals of nickel-cobalt sulfide (Ni_3_Co_6_S_8_) uniformly distributed on a crumpled rGO structure (Figure 2A) via spray pyrolysis was prepared as an anode for SIBs. The small Ni_3_Co_6_S_8_ plate-shape nanocrystals were embedded in rGO, resulting in a 3D hollow interconnected nanocomposite (Figure 2B) (Choi and Kang, 2015a). In addition, a yolk–shell-structured (Fe_0.5_Ni_0.5_)_9_S_8_ solid-solution powder was prepared by the same group via a one-pot spray pyrolysis process as anode for SIBs. As a result, an excellent electrochemical performance was achieved. Schematic diagrams of the preparation process are shown in Figures 2C,D (Kim and Kang, 2017).

The primary advantage of this method is that BMS electrode materials could be synthesized in only one step, and without any further treatments.

### Co-precipitation Method

The co-precipitation method has been used in recent years for preparing homodisperse BMSs nanostructure materials in SIBs. This method is proved to possess prominent virtues, such as easily obtaining nanomaterials with high phase purity, and preparing nano-powder with controllable particle size and uniform distribution.

By employing the co-precipitation method, Yang et al. reported a kind of (SnCo)S_2_/rGO nanocubes (Figure 2E) (Yang et al., 2019). In addition, Ou and his co-workers synthesized MnSn(OH)_6_ nanoboxes firstly through a straightforward co-precipitation process, then SnS_2_/Mn_2_SnS_4_/C nanoboxes (SMS/C) were prepared through the facial wet-chemical method. As an anode material for SIBs, the SMS/C electrode can show a high ICE of 90.8%, excellent rate capability (488.7 mAh g^−1^ at 10 A g^−1^) and long cycling stability (522.5 mAh g^−1^ at 5 A g^−1^ was retained after 500 cycles) (Ou et al., 2019).

Due to its advantages, an easy operation, low cost, and less synthesis time, the co-precipitation method has been broadly utilized to prepare BMSs as anode materials for SIBs.

### Other Methods

**Figure 3:**
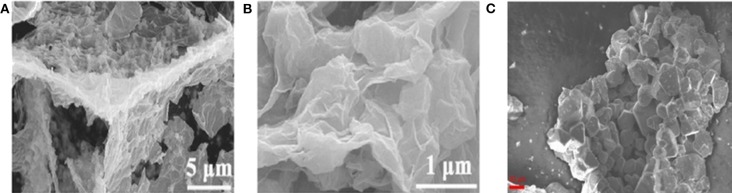
**(A,B)** SEM images of [Zn_x_Co_1−x_S QD@HCP]@rGO composites. Reproduced with permission from Chen Z. et al. (2017) Copyright 2017. Wiley-VCH. **(C)** SEM image of CuV_2_S_4_. Reproduced with permission from Krengel et al. (2017) Copyright 2017. American Chemical Society.

In addition to the mentioned-above synthesis methods, an increasing number of high-efficiency ways have been explored to prepare BMSs with different structures. For instance, sponge-like composite of (ZnxCo_1−x_S QD@HCP)@rGO were reported by Sun's group through a simultaneous thermal-induced sulfidation, carbonization, and reduction. The as-obtained ZnxCo_1−x_S quantum dots (QD) were uniformly distributed on mesoporous hollow carbon polyhedral (HCP) matrix and rGO coating with large specific surface, denoted as [ZnxCo_1−x_S QD@HCP]@rGO (Figures 3A,B) (Chen Z. et al., 2017; Hwang et al., 2017). By using the solid state reaction method, Krengel synthesized CuV_2_S_4_ particles with a broad size distribution between 5 and 50 μm (Figure 3C). The obtained products delivered an excellent cycling stability of 580 mAh g^−1^ was retained after 500 cycles at 0.7 A g^−1^ and relatively high ICE of 72.5% (Qin et al., 2016a; Xu et al., 2016; Zhou J. et al., 2016; Krengel et al., 2017).

With the novel methods developing to synthesize nanomaterials with unique structures, a plenty of BMSs with high-efficiency nanostructure have been applied in EES. A comparison of the structural properties, synthesis methods, and S resource of BMSs is summarized in Table 2.

**Table 2:** Comparison between the structural properties, synthesis methods, and S resource of BMSs.

**Materials**	**Morphology & Size (nm)**	**Synthetic method**	**S resource**
NiCo_2_S_4_@NC	Nanoparticles 9	Hydrothermal	TAA
Ni_3_Co_6_S_8_-rGO	Plate-shape nanocrystals —	Ultrasonic spray pyrolysis	Thiourea
NiCo_2_S_4_-rGO	Hollow prism 500–600	Refluxing & solvothermal	TAA
Co_1_Zn_1_-S (600)	Nanocrystals —	Oil-bath & thermally treated	TAA
Co_8_FeS_8_	Nanocubes 100	Wet chemical bath	Thiourea
Co_0.5_ Fe_0.5_S_2_	Nanosphere ~100	Solvothermal	Sublimed S
(Zn_x_Co_1−x_S QD@HCP) @rGO	sponge-like —	Thermal-induced sulfidation, carbonization, and reduction reaction	Sublimed S
(SnCo)S_2_/SG	Nanocubes 300–400	annealing	Sublimed S
(Ni_0.3_Co_0.7_)_9_S_8_/N-CNTs/rGO	Nanoparticles —	Coprecipitation/Hydrothermal	TAA
CuCo_2_S_4_/rGO	Nanoparticles 10–50	Solvothermal	Thiourea
Ti_0.25_Sn_0.75_S_2_@MWCNTs	Lantern-like 750–850	Hydrothermal	TAA
CoSnS_x_@NC	Nanoboxes ~150	Solvothermal	TAA
N/S-rGO @ZnSnS_3_	Hollow Nano-microcubes ~2,000	Coprecipitation/Hydrothermal	Na_2_S/ Thiourea
Materials	Morphology & Size (nm)	Synthetic method	S resource
VMo_2_S_4_-rGO	Nanosheet —	Solvothermal	TAA
ZnSnS_3_@ rGO	Nanoparticles —	Solvothermal & Annealing	Thiourea
(Co_0.5_Ni_0.5_)_9_S_8_/NC	Nanoparticles & Nanorods —	Solvothermal	Sulfur powder
Cu_2_MoS_4_	Nanoparticles 100	Solvothermal	TAA
SnS_2_/Mn_2_SnS_4_/C	Nanoboxes 100	Coprecipitation	Sulfur powder
CuCo_2_S_4_	Sub-microspheres 300–500	Solvothermal	Thiourea
NiMo_3_S_4_/CT	Nanosheets arrays 3,000	Hydrothermal	Thiourea
(Fe_0.5_Ni_0.5_)_9_S_8_	Yolk-shell —	Spray pyrolysis	Thiourea
Bi_0.94_Sb_1.06_S_3_	Nanorod cluster	Hydrothermal	TAA
CuV_2_S_4_	Nano-polyhedron	Solid state reaction	Sulfur powder

As mentioned, the nanomaterials obtained by a solvothermal method are characterized by good crystal morphology, controllable nanometer size, and high purity. However, it may be difficult to scale up the production. The spry pyrolysis results in the powder materials with the merits of small nanometer size and uniform dispersion, but this promising method needs some special equipment with complex operation. Despite some advantages of easy operation, low-cost and shorter reaction time, co-precipitation method still cause some challenges to solve, for example, the reaction rate is not controllable, with the server agglomeration of nanomaterials. Therefore, the desired and materials may be considered through choosing suitable synthesis strategies for BMSs (Lai et al., 2012; Palomares et al., 2012).

## Applications in SIBS

### Transition BMSs

Considering the specific reaction mechanism, abundant active sites and short diffusion path-ways, transition BMSs nanomaterials have many advantages as promising anode materials for SIBs. A large amount of work has been devoted to the development of transition BMSs anodes in SIBs. In this section, transition BMSs as high-performance SIB anode materials are discussed and reviewed.

**Figure 4:**
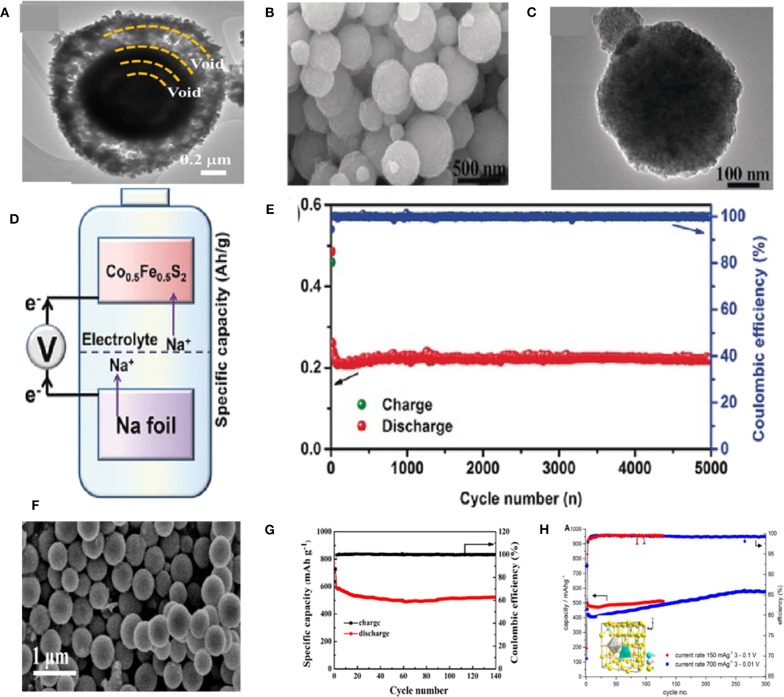
**(A)** TEM images of the (Fe_0.5_Ni_0.5_)_9_S_8_ yolk–shell powder. Reproduced with permission from Kim and Kang (2017) Copyright 2017, Tsinghua University Press. **(B,C)** SEM and TEM images of the Co_0.5_Fe_0.5_S_2_ sample. **(D,E)** Illustration of the composition and cycling performance of Na/Co_0.5_Fe_0.5_S_2_ half-cell. Reproduced with permission from Zhang et al. (2016) Copyright 2016, Wiley-VCH. **(F)** SEM image of the CuCo_2_S_4_ sub-microspheres; **(G)** Cycling performance of CuCo_2_S_4_. Reproduced with permission from Li Q. et al. (2019) Copyright 2019, Wiley-VCH. **(H)** Cycling performance and coulombic efficiency of CuV_2_S_4_ cells, using galvanostatic cycling at 0.15 A g^−1^ between 3 and 0.1 V and 3 and 0.01 V at 0.7 A g^−1^. The inset in **(H)** shows the spinel type unit cell. Reproduced with permission from Krengel et al. (2017) Copyright 2017, American Chemical Society.

In some case, a yolk–shell-structured Fe–Ni–O was designed via one-pot spray pyrolysis as shown in Figure 4A. When employed as an anode in SIBs, (Fe_0.5_Ni_0.5_)_9_S_8_ exhibited a capacity of 527 mAh g^−1^ at 1 A g^−1^ after 100 cycles. An outstanding rate performance was also obtained with a reversible discharge capacity of 465 mAh g^−1^ at 5.0 A g^−1^ (Kim and Kang, 2017). Kang et al. investigated cobalt doped FeS_2_ through changing the content of Co by a simple solvothermal method. When employed as an anode material in SIBs for the first time, the Co doped FeS_2_ showed a good cycling and rate performance in a voltage range of 0.8–2.9 V owing to the high rate capability of FeS_2_ and high capacity of CoS_2_. All samples displayed spherical particle shapes with average diameters of about 100 nm (Figures 4B,C). When Co contents increased to 0.5, Co_0.5_Fe_0.5_S_2_ showed the best electrochemical performances. As shown in Figures 4D,E, a stable specific capacity of 220 mAh g^−1^ was achieved after 5,000 cycles at 2 A g^−1^ (Zhang et al., 2016; Ge et al., 2017). Feng et al. used a simple solvothermal method to synthesize CuCo_2_S_4_ sub-microspheres with the sizes ranged from 300 to 500 nm (Figure 4F). The unique structure and dual metal synergistic effects of CuCo_2_S_4_ can effectively improve the stability of electrode materials by avoiding aggregation of nanomaterials and shortening the ion/electron diffusion pathways. The obtained CuCo_2_S_4_ composite displayed an excellent cycling stability and high coulombic efficiency as anode for SIBs Figure 4G (Li Q. et al., 2019). As depicted in the inset of Figure 4H, an irregular micro-polyhedron CuV_2_S_4_ was synthesized via a solid state reaction method. The cycling capability of the CuV_2_S_4_ as shown in Figure 4H, which displays a capacity of 490 mAh g^−1^ at 0.15 A g^−1^ and 410 mAh g^−1^ at 0.7 A g^−1^. The intermediate product Na_2_S matrix starts to participate in the redox process causing a stable capacity increase up to 580 mAh g^−1^ during the first 250 cycles at 0.7 A g^−1^ and maintaining it at this level during the next 50 cycles (Krengel et al., 2017).

In conclusion, the abundance of transition metals with different valence states makes them exhibit a high theoretical specific capacity during the electrochemical reactions.

Despite the many advantages of BMSs, the challenges still remain in terms of sluggish reaction kinetics, bad electrochemical properties due to the large radius of Na^+^ and considerable volume-change during the cycling process. To overcome the pitfalls mentioned above, carbon-based materials were introduced because of their cycling stability, wide-abundant resources, and low sodium embedded platform. Indeed, coating and doping BMSs with carbon materials have been employed as promising methods for enhancing the sodium ions storage performance in SIBs because they can improve the electroconductivity and maintain the structural stability of BMSs (Chen S. et al., 2017; Lin et al., 2018; Lv et al., 2018; Zhang et al., 2018).

**Figure 5:**
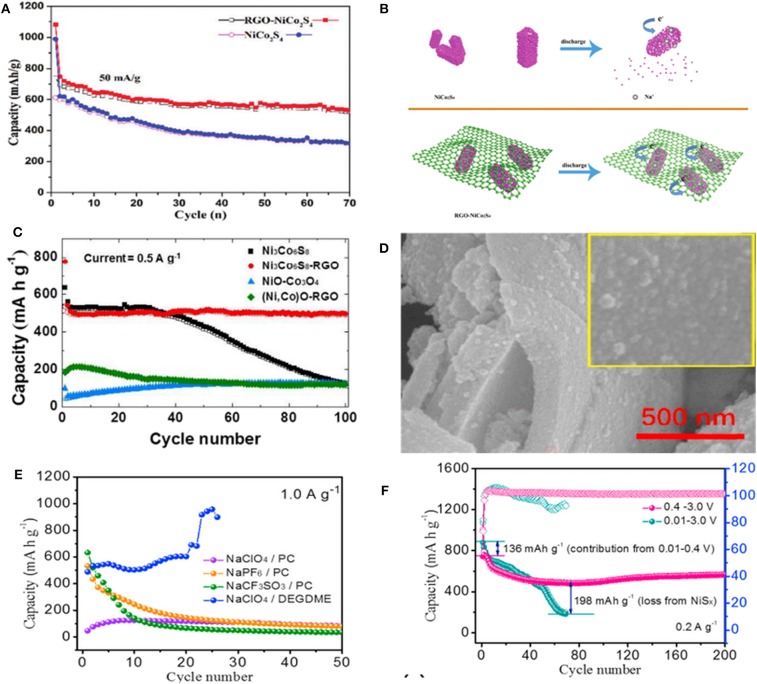
**(A)** The cycling performance of NiCo_2_S_4_ and rGO–NiCo_2_S_4_ at 50 mA g^−1^. **(B)** Schema of Na-ion insertion process in NiCo_2_S_4_ and rGO–NiCo_2_S_4_. Reproduced with permission from Zhang et al. (2018) Copyright 2018. Royal Society of Chemistry. **(C)** Cycling performances of (Ni,Co)O-rGO and Ni_3_Co_6_S_8_- rGO at 0.5 A g^−1^. Reproduced with permission from Choi and Kang (2015a) Copyright 2015. Royal Society of Chemistry. **(D)** SEM images of NiCo_2_S_4_-NC, **(E)** Cycling performances of NiCo_2_S_4_-NC in different electrolytes at 1.0 A g^−1^, **(F)** Cycling performance and coulombic efficiency of NiCo_2_S_4_-NC in different cut-off voltage windows at 0.2 A g^−1^. Reproduced with permission from Li S. et al. (2019) Copyright 2019. Elsevier.

As a typical BMS, NiCo_2_S_4_ has drawn much attention due to its excellent electroconductivity, extremely stable electrochemical cycling performance and outstanding rate capability. Nonetheless, its sluggish Na^+^ kinetics has limited the advancement of this anode material. To overcome this issue, the composites of NiCo_2_S_4_ with C-based materials, such as N-doped carbon (NC), rGO and carbon nanotubes (CNTs) have been investigated. The carbon-based materials can not only improve the electroconductivity, but also provide more active sites for fast Na^+^ storage and alleviate the volume expansion during the charge-discharge process (Xiao et al., 2017). For instance, Yin et al. reported the effectiveness of rGO matrix in enhancing electrochemical properties of NiCo_2_S_4_ hollow prism confirmed by its cycling performance (Figure 5A). During the discharge process, the hollow NiCo_2_S_4_ shell nanoparticles will be collapsed when Na^+^ inserts into the anode, while the NiCo_2_S_4_ nanomaterial wrapped in rGO can be well-preserved (Figure 5B) (Zhang et al., 2018). Therefore, ultrathin rGO nanosheets with large specific surface area, active site and porous channels lead to an outstanding electrochemical performance with a well Na-storage. Figure 5C illustrates the cycling performance of Ni_3_Co_6_S_8_@rGO electrode at 0.5 A g^−1^ prepared by Kang et al. with distributing the plate-shape nanocrystals of Ni_3_Co_6_S_8_ over the crumpled rGO structure. These nanocrystals delivered a capacity of 298.1 mAh g^−1^ after 300 cycles at 25 mAh g^−1^ as anode material in SIBs (Choi and Kang, 2015b). CuCo_2_S_4_/rGO nanocomposites were synthesized, which displayed a capacity of 433 mAh g^−1^ after 50 cycles at 0.1 A g^−1^ and delivered an excellent rate performance with 336 mAh g^−1^ at 1 A g^−1^ (Gong et al., 2018).

Moreover, combining with graphene, Ji et al. utilized a bottom-up strategy for preparing NiCo_2_S_4_ nanodots uniformly incorporated with N-doped carbon (denoted as NiCo_2_S_4_-NC) (Figure 5D). Then, the influence of different electrolytes and voltage windows on its electrochemical performance was investigated. As shown in Figure 5E, due to the flexible one-dimensional chain structure of DEGDME, the cell with the ether-based NaClO_4_/DEGDME electrolyte delivered the highest capacity of 530 mAh g^−1^ at 1.0 A g^−1^. Indeed, the best voltage range was realized to be 0.4–3.0 V, in which the cell can effectively maintain a reversible phase transformation and avoid the side reaction (Figure 5F) (Li S. et al., 2019). Chen et al. also synthesized N-doped carbon coated Co_8_FeS_8_ hollow nanocubes with large surface area, small charge transfer resistance, and fast Na^+^ diffusion coefficient. Moreover, a layered Cu_2_MoS_4_-rGO with crystal structure was prepared by this group (Chen et al., 2019).

**Figure 6:**
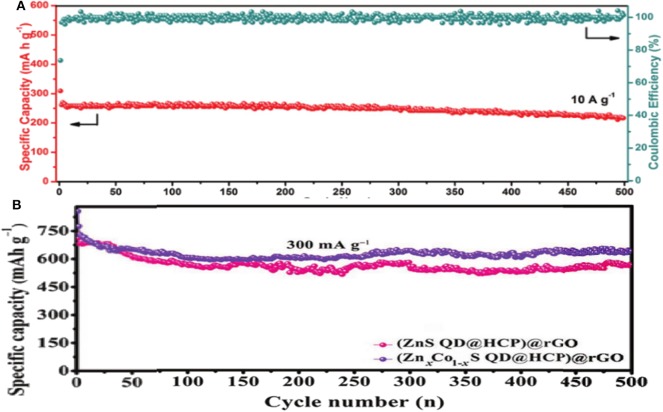
**(A)** Cycling performance of Co_1_Zn_1_-xS. Reproduced with permission from Fang G. et al. (2018) Copyright 2018. Wiley-VCH. **(B)** Cycling performance of (ZnS QD@HCP)@rGO and (Zn_x_Co_1−x_S QD@HCP)@rGO composites at 3 A g^−1^. Reproduced with permission from Chen Z. et al. (2017) Copyright 2017. Wiley-VCH.

Co_1_Zn_1_-xS(600) is another unique composite structure prepared through a simple sulfidation and calcination. This special structure can retard the volume-change during the electrochemical process, expediate the Na^+^ diffusion kinetics and enhance the electroconductivity, leading to a relatively low irreversible capacity, and superior cycling and rate performance (Figure 6A). When used in SIBs, an excellent capacity of 542 mAh g^−1^ can be achieved after 100 cycles at 0.1 A g^−1^, with an impressive rate property of 219.3 mAh g^−1^ at 10 A g^−1^ (Choi et al., 2015; Qin et al., 2016b; Fang G. et al., 2018; Wang et al., 2018). In another study, a sponge-like (Zn_x_Co_1−x_S QD@HCP)@rGO composite combined with mesoporous hollow carbon polyhedral (HCP) matrix and rGO wrapped sheets was prepared. Due to the merits of this structure, (Zn_x_Co_1−x_S QD@HCP)@rGO as a binder-free anode in SIBs showed a good reversible capacity and cycling performance (i.e., 638 mAh g^−1^ at 0.3 A g^−1^ after 500 cycles), which was better than that of the monometallic sulfide under the same conditions (Figure 6B) (Chen Z. et al., 2017). In order to solve the problems of low energy density and poor cycle life when used as an anode in SIBs, MOF precursors were utilized to fabricate *in-situ* NC embellished with BMS hollow spheres nanomaterials. They prepared (Co_0.5_Ni_0.5_)_9_S_8_ solid-solution combined with *in-situ* NC [donated as (Co_0.5_Ni_0.5_)_9_S_8_/NC], which exhibited a superior Na-storage properties. Indeed, a good specific capacity of 723.7 mAh g^−1^ was maintained after 100 cycles at 1 A g^−1^, with 83% coulombic efficiency compared to the second cycle. The impressive rate capability of 596.1 mAh g^−1^ was achieved at 10 A g^−1^ with a high capacity retention of 60.2% at 0.1 A g^−1^, demonstrating an excellent rate performance. As a result of carbon modification and the hierarchical sphere structures, high electrical conductivity, and mechanical stability were achieved during the cycling process (Cao et al., 2019).

Because of the inherent disadvantages of BMSs, the electrode materials are highly susceptible to expansion and then easily detached from the current collector during the cycling. The related results demonstrated that carbon modification and optimization of the nanostructure are good choices to obtain high performance sodium ions storage system. In addition, Yang et al. designed a bind-free electrode material as SIBs anode, which has a NiMo_3_S_4_/CTs nanosheet arrays with hierarchical hybrid nanostructure (Kong et al., 2018). Consequently, it delivered a high sodium storage capacity and an excellent cycling performance.

In the past decade, a large amount of studies has been done to explore excellent electrode materials for Na-storage. Hence, a detailed comparison of the electrochemical performance of BMS anodes in SIBs are presented in Table 3.

**Table 3:** Comparison between the electrochemical performance of BMS anodes in SIBs.

**Materials**	**Current density [mA g^**−1**^]**	**Cycle number**	**Cut-off voltage [V]**	**Specific capacity [mA h g^**−1**^]**
NiCo_2_S_4_@NC	100 6,000	200 5,000	0.4–3	570.1 395.6
	100	1	0.4–3 0.01–3	742.2 878.2
		70	0.4–3 0.01–3	570.1 181.3
Ni_3_Co_6_S_8_-RGO	500	2 100	0.001–3	504 498
Ni_3_Co_6_S_8_		2 100		522 125
NiCo_2_S_4_@RGO	50	20 70	0.01–3	603.5 530.2
NiCo_2_S_4_		20 70		462.1 317
Co_1_Zn_1_-S (600)	100	1 100	0.01–3	745 542
	10,000	5,000		219.3
Co_8_FeS_8_	50	40	0.01–3	≈500
	500	150		87
Co_0.5_ Fe_0.5_S_2_	100	60	0.8–2.9	328
	2,000	5,000		220
(Zn_x_Co_1−x_S QD@HCP) @rGO	300	500	0.01–2.9	638
(SnCo)S_2_/SG	500	300	0.01–3	593
	5,000	5,000		487
(Ni_0.3_Co_0.7_)_9_S_8_/N-CNTs/rGO	25	300	0.01–3	298.1
CuCo_2_S_4_/rGO	100	50	0.01–2.5	433
Ti_0.25_Sn_0.75_S_2_ @MWCNTs	200	50	0.01–2.5	307
	400	1,000		388
CoSnS_x_@NC	200	500	0.01–3	300
	1,000	4,000		180
N/S-rGO@ZnSnS_3_	100	100	0.01–3	501.7
	1,000	500		290.7
VMo_2_S_4_-rGO	1,000	200	0.01–3	254
ZnSnS_3_@ rGO	100	200	0.01–2.5	401.2
(Co_0.5_Ni_0.5_)_9_S_8_/NC	50	100	0.01–3	569.9
Cu_2_MoS_4_	500	2,000	0.5–3	205.7
SnS_2_/Mn_2_SnS_4_/C	5,000	500	0.1–3	522.5
CuCo_2_S_4_	200	140	0.01–3	522.4
NiMo_3_S_4_/CT	480	1,000	0.01–3	302
(Fe_0.5_Ni_0.5_)_9_S_8_	1,000	100	0.01–3	527
Bi_0.94_Sb_1.06_S_3_	1,000	100	0.01–2.8	380
CuV_2_S_4_	150	150	0.1–3	500
	700	300	0.01–3	580

### Mixed BMSs

Tin-based BMSs (ZnSnS_3_, CoSnS_x_) have shown high capacity as SIB anodes, and attracted extensive attention due to the large interlayer spacing originated from their CdI2-type layered structure, and high theoretical capacity owing to the combination of conversion and alloying types electrochemical reaction mechanism (Qu et al., 2014; Choi et al., 2015; Cho et al., 2016; Lu et al., 2016). However, it is essential to resolve the problems rooted in large volume expansion and their low conductivity. Therefore, structure engineering and introduction of carbon materials have been sought to change the electrochemical properties of BMSs.

**Figure 7:**
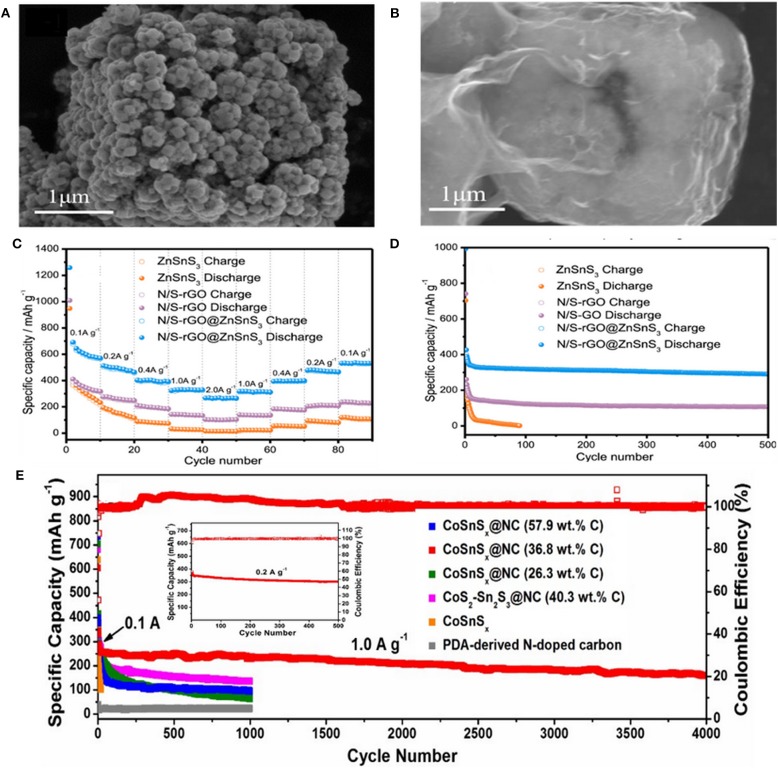
**(A,B)** FESEM images of ZnSnS_3_ and N/S-rGO@ZnSnS_3_, **(C,D)** Rate and cycling performance of N/S-rGO, ZnSnS_3_, and N/S-rGO@ZnSnS_3_ electrodes. Reproduced with permission from Liu et al. (2019) Copyright 2019. Elsevier. **(E)** Long-term stability of amorphous CoSnS_x_@NC nanoboxes with various carbon content, amorphous CoSnS_x_ nanoboxes, crystalline CoS-Sn_2_S_3_@NC nanoboxes and PDA-derived N-doped carbon at 1.0 A g^−1^. The inset in **(E)** shows the cycling performance and coulombic efficiency of the CoSnS_x_@NC nanoboxes electrode at 0.2 A g^−1^. Reproduced with permission from Liu et al. (2017) Copyright 2017. Royal Society of Chemistry.

Zinc tin sulfide@rGO (ZnSnS_3_@rGO) nanoparticles were prepared by Zhang et al. via combining solvothermal reaction with the annealing process. When used in SIBs, superior Na-storage performance with large specific capacity (472.2 mAh g^−1^ at 0.1 A g^−1^), high rate capability (165.8 mAh g^−1^ at 2 A g^−1^), and ultralong cycle life (401.2 mAh g^−1^ at 0.1 A g^−1^ after 200 cycles) was achieved (Jia et al., 2018). Therefore, the introduced composite anode design provides new changes for development of highly stable anode materials which possess excellent conductivities and high adaptability for large volume-changes during the sodiation/desodiation process. Liu et al. designed ZnSnS_3_ nanostructure with hollow nano-microcubes through co-precipitation and hydrothermal methods. The process was followed by coating the N/S dual-doped rGO (N/S-rGO@ ZnSnS_3_) (Figures 7A,B) to enhance the slow reaction kinetics, poor electrochemical properties of the BMS. As a result, the prepared N/S-rGO@ ZnSnS_3_ composite exhibited a high specific capacity of 501.7 mAh g^−1^ after 100 cycles at 0.1 A g^−1^ and an excellent long cycle life of 290.7 mAh g^−1^ after 500 cycles at 1 A g^−1^. Meanwhile, a high-rate capacity of 256.6 mAh g^−1^ at 2 A g^−1^ was maintained (Figures 7C,D). Such outstanding performances were primarily ascribed to the coating of dual-doped rGO which provides some synergetic merits for EES as follows: (1) due to the strong polarity of the doping area that restraining the aggregation of the prepared rGO; (2) enhancing the electroconductivity by reducing the semiconducting gap; (3) because of the deficiencies possess high-electronegativity can easily attract the positive ions that leading to increase the number of alkali metal ions; (4) owing to the adsorption effect among anode and rGO that reinforcing the structural stability (Liu et al., 2019). In addition, Chen et al. introduced titanium into the crystal structure of SnS_2_ to partially replace tin, forming the lantern-like Ti_0.25_Sn_0.75_S_2_ followed by coating one-dimensional multi-walled carbon nanotubes (MWCNTs) (denoted as Ti_0.25_Sn_0.75_S_2_@MWCNTs) to improve the defects of SnS_2_ volume expansion and low conductivity. Profiting from its lantern-like structure with a large specific surface area, the electrolyte could fully infiltrate into Ti_0.25_Sn_0.75_S_2_@MWCNTs, increasing the electron/ion transfer during cycling. A high specific capacity of 307 mAh g^−1^ was acquired after 1,000 cycles at 0.4 A g^−1^ during the electrochemical testing process (Huang et al., 2018). The single-crystal mesoporous CoSn(OH)_6_ nanoboxes were also synthesized through co-precipitation. TAA was employed as S resource to reach CoSnSx via solvothermal method, followed by polymer nanoplating and carbonization with dopamine at higher temperature in N_2_ flow to obtain CoSnS_x_@NC electrode materials. Subsequently, the Na-storage performance and influence of carbon content on the electrochemical properties of CoSnS_x_@NC nanoboxes were investigated. The results indicated that the best amount of carbon content is 36.8 wt.% to protect the nanoboxes from destruction during deep cycling. The electrode exhibited an excellent cycling performance and achieved a high capacity of 300 mAh g^−1^ with a high coulombic efficiency of nearly 100% after 500 cycles, as well as an outstanding long-life cycling of 180 mAh g^−1^ after 4,000 cycles at 1 A g^−1^ (Figure 7E) (Liu et al., 2017). Moreover, Ou et al. prepared heterostructured SnS_2_/Mn_2_SnS_4_/carbon nanoboxes with about 100 nm in size through a facial co-precipitation method. When evaluated as an anode material in SIBs, the special structure between SnS_2_ and Mn_2_SnS_4_ can alleviate the volume change upon a mass of electrochemical process, prevent the cohesion of Sn nanoparticles, and boost the reversibility of conversion-alloying reaction. It also demonstrated a high ICE of 90.8%, an outstanding long cycling stability of 522.5 mAh g^−1^ after 500 cycles at 5 A g^−1^, and a remarkable rate capability (752.3, 604.7, 570.1, 546.9, 519.7, and 488.7 mAh g^−1^ at 0.1, 0.5, 1.0, 2.0, 5.0, and 10.0 A g^−1^, respectively). Benefitting from these advantages (huge specific surface area, abundant active sites, and strong electrical conductivity) of carbon materials, the resulting composite electrode displayed an impressive electrochemical performance (Ou et al., 2019). Yang et al. reported a novel material consisted of (SnCo)S_2_ nanocubes interlaced with 2D sulfur-doped graphene (SG) nanosheets ((SnCo)S_2_/SG) synthesized by a simple co-precipitation method and annealing. It exhibited an excellent reversible capacity of 487 mAh g^−1^ for 5,000 cycles at 5 A g^−1^ as well as a high capacity retention of 92.6% (Yang et al., 2019).

### Other BMSs

**Figure 8:**
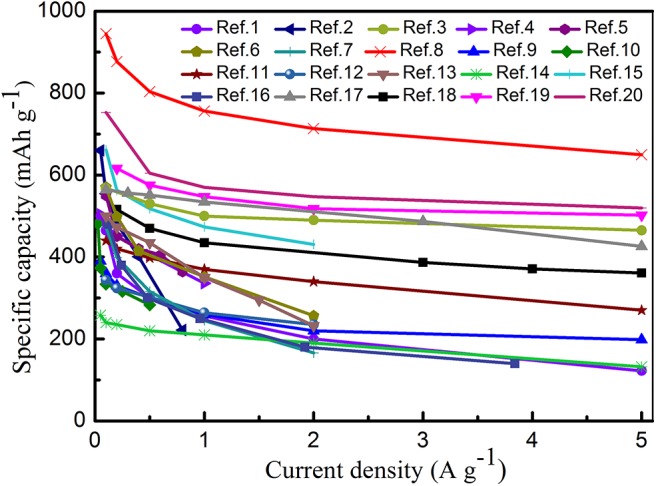
Rate capability at various current densities from 0.1 to 5 A g^−1^ for different bimetallic sulfides in SIBs. Ref.1 (Choi and Kang, 2015a), Ref.2 (Chen J. et al., 2017), Ref.3 (Zhang et al., 2016), Ref.4 (Yang et al., 2019), Ref.5 (Lv et al., 2018), Ref.6 (Zhang et al., 2018), Ref.7 (Gong et al., 2018), Ref.8 (Huang et al., 2018), Ref.9 (Liu et al., 2017), Ref.10 (Liu et al., 2019), Ref.11 (Zhang K. et al., 2019), Ref.12 (Jia et al., 2018), Ref.13 (Cao et al., 2019), Ref.14 (Chen et al., 2019), Ref.15 (Ou et al., 2019), Ref.16 (Li Q. et al., 2019), Ref.17 (Kong et al., 2018), Ref.18 (Kim and Kang, 2017), Ref.19 (Zhao and Manthiram, 2015), Ref.20 (Krengel et al., 2017).

In addition to the above-mentioned BMSs, Manthiram et al. have reported a nano-rod cluster Bi_0.94_Sb_1.06_S_3_-graphite as SIB anode material. They found that the design of solid solutions can be regarded as an ideal method to research new anode materials with excellent electrochemical performances for SIBs. The Bi_0.94_Sb_1.06_S_3_-graphite anode displayed a remarkable capacity of 380 mAh g^−1^ after 200 cycles at 1 A g^−1^, which is higher than the that of Sb_2_S_3_-graphite electrode (~50 mAh g^−1^) and Bi_2_S_3_-graphite electrode (~210 mAh g^−1^). This means that bimetallic atoms can not only enhance the cycling stability of electrode materials, but also improve their capacity (Zhao and Manthiram, 2015). Zhong et al. successfully designed a novel yolk-shell hydrangea-like micro-flower composite self-assembled by nanosheets for SIBs. Accordingly, a high capacity of 607.14 mAh g^−1^ was delivered at 0.05 A g^−1^, along with reducing the volume expansion and enhancing the cycling stability to a great extent because of the unique structure of the electrode material (Zhong et al., 2019). Furthermore, the rate performance of different BMS materials is depicted in Figure 8, and a comparison between the cycle performances of BMSs and MSs is summarized in Table 4.

**Table 4:** Comparison between the electrochemical performance of BMS and MS anodes in SIBs.

**BMSs**	**MSs**	**Current density (A g^**−1**^)**	**Cycle number**	**Specific capacity (mAh g^**−1**^)**
	CoS_2_/C	0.5	60	330
NiCo_2_S_4_@NC		6	5,000	395.6
	NiS_2_	0.5	1,000	319
	FeS_2_	1	400	180
Co_0.5_Fe_0.5_S_2_		2	5,000	220
	Co_9_S_8_	1	70	266
	SnS_2_-rGO	1	400	500
(SnCo)S_2_/SG		5	5,000	487
	—	—	—	—
	Co_9_S_8_/NC	0.05	100	274.7
(Co_0.5_Ni_0.5_)_9_S_8_/NC				723.7
	Ni_3_S_2_/NC			601.7
	SnS_2_/C	5	500	235
SnS_2_/Mn_2_SnS_4_/C				522.5
	—	—	—	—
	MoS_2_-rGO	1	200	209
VMo_2_S_4_-rGO				254
	—	—	—	—
	MoS_2_/CTs	0.48	500	135
NiMo_3_S_4_/CTs			1,000	302
	NiS_x_/CTs		500	61
	FeS	1	80	351
(Fe_0.5_Ni_0.5_)_9_S_8_				601
	NiS			293
	Sb_2_S_3_-G	1	200	80
Bi_0.94_Sb_1.06_S_3_-G				380
	Bi_2_S_3_-G			220

## Conclusions and Perspectives

In this review, the latest development of BMSs as anode materials for SIBs was systematically summarized. BMSs reveal obvious merits with relatively high electroconductivity and electrochemical activity. Moreover, the significant effect of self-matrix and self-conductivity due to the reaction of two metal elements with Na can be totally effective. Indeed, due to the existence of “synergistic effect,” the non-reacted part can serve as a provisional snubber/conductor for the reacted one owing to their different redox potential (Pumera et al., 2014; Wang et al., 2014; Chang et al., 2016; Liu et al., 2019). In this review, firstly, the synthesis strategies of BMSs have been introduced. Then, Na-storage mechanisms of various BMSs during the charge-discharge process has been discussed. More importantly, BMSs application as SIB anodes has been systematically analyzed, while putting forward insightful anticipations on its future development.

To avoid capacity fading of the BMS anode materials, the first strategy is designing novel nanostructures with suitable void space to alleviate the influence of volume expansion and contraction during the reaction process (Palomares et al., 2012; Slater et al., 2013; Ou et al., 2016; Putungan et al., 2016; Shen et al., 2016; Su et al., 2016). As the second strategy, integration with other electrochemically stable materials can not only restrain the volume expansion, but also advance the overall electroconductivity of the anode. Additionally, the dissolution of polysulfides in electrolyte during the electrochemical process can be suppressed to some extent (Wang et al., 2018). Up to now, plenty of reported BMS anode in SIBs refer to their combination with carbon-based materials. Thus, it is important for the development of SIBs anode materials to fully study the merits of nanostructured materials (Lu et al., 2017; Ma et al., 2018). In the future, much more efforts need to be made to overcome the disadvantage of poor long-term cycling. It is expected that exploitation of rational-designed structures in BMSs can effectively improve the electrochemical performance in SIBs (Kim et al., 2012; Jiang et al., 2014; Su et al., 2015; Gao et al., 2017; Hwang et al., 2017).

Despite the all novel work has been done thus far, still more time, and efforts should be dedicated to effectively boost the electrochemical properties of BMSs to pave the way of their practical application in SIBs in the near future.

## Author Contributions

YH, DX, and XL contributed to the conception and design of the study. YH organized the database, performed the statistical analysis, and wrote the manuscript with the help of HM, JP, YiL, YuL, DL, QS, and XS. All authors have given approval to the final version of the manuscript.

## Conflict of Interest

The authors declare that the research was conducted in the absence of any commercial or financial relationships that could be construed as a potential conflict of interest.
